# Methanol‐to‐Olefins in a Membrane Reactor with in situ Steam Removal – The Decisive Role of Coking

**DOI:** 10.1002/cctc.201901222

**Published:** 2019-11-25

**Authors:** Felix Rieck genannt Best, Alexander Mundstock, Gerald Dräger, Pascal Rusch, Nadja C. Bigall, Hannes Richter, Jürgen Caro

**Affiliations:** ^1^ Institute for Physical Chemistry and Electrochemistry Leibniz University Hannover Callinstraße 3 A Hannover 30167 Germany; ^2^ Institute for Organic Chemistry Leibniz University Hannover Schneiderberg 1B Hannover 30167 Germany; ^3^ Institute for Ceramic Technologies and Systems Fraunhofer IKTS Michael-Faraday-Straße 1 Hermsdorf 07629 Germany

## Abstract

The reaction of methanol to light olefins and water (MTO) was studied in a fixed bed tubular membrane reactor using commercial SAPO‐34 catalyst. In the fixed bed reactor without membrane support, the MTO reaction collapsed after 3 h time on stream. However, if the reaction by‐product steam is in situ extracted from the reactor through a hydrophilic tubular LTA membrane, the reactor produces long‐term stable about 60 % ethene and 10 % propene. It is shown that the reason for the superior performance of the membrane‐assisted reactor is not the prevention of catalyst damage caused by steam but the influence of the water removal on the formation of different carbonaceous residues inside the SAPO‐34 cages. Catalytically beneficial methylated 1 or 2 ring aromatics have been found in a higher percentage in the MTO reaction with a water removal membrane compared to the MTO reaction without membrane support.

## Graphical abstract

The Methanol‐to‐Olefins (MTO) reaction was performed in a membrane reactor with in situ steam removal through on tubular LTA zeolite membrane, where it showed exceptional long‐term stability over 4 hours, while maintaining a stable product distribution. Further examination showed a different coking behavior as the reason for the improvement compared to a fixed bed reactor without membrane support.

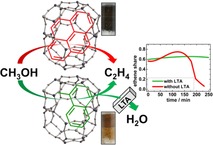

## Introduction

The majority of light olefins as the basic of our today's chemical industry is produced via steam cracking. Increasingly, alternative ways of light olefin formation are studied.^1^, ^2^, ^3^ The methanol‐to‐olefins (MTO) process, which is based on the catalytic conversion of methanol to light olefins and water, offers a new and eco‐friendly synthesis route. Methanol as the feedstock can be easily produced by catalytic conversion of synthesis gas, which is usually provided by methane (natural or biogas) steam reforming and/or partial oxidation as well as directly from coal. Different acidic molecular sieve catalysts like zeolites or aluminum phosphates such as SAPO‐34,^4^ ZSM‐5^5^ or ZSM‐22^6^ have been evaluated in the MTO process and show decent selectivities towards the light olefins ethene and propene. Alkenes with higher molecular weights, methane and other paraffins are the main by‐products of the MTO reaction. However, the product composition heavily depends on the reaction parameters like particle sizes, temperature, time on stream (TOS) and catalyst cage dimensions.^7^, ^8^, ^9^, ^10^, ^11^ The exact mechanism, in particular the forming of the first C−C bond from methanol, is still subject of uncertainty. Dahl et al. first introduced the mechanism of the hydrocarbon pool (HCP) in 1993,^12^ which is researched extensively and extended since.^13^, ^14^, ^15^, ^16^, ^17^ Several studies led to the conclusion that aromatic species in the HCP take part in the formation of light olefins due to de‐ and re‐alkylation of the aromatic rings rather than by direct dimerization of methanol. MTO as an autocatalytic reaction is fueled by the formation of those beneficial aromatics in the HCP, which leads to an increased methanol conversion.^18^ However, these aromatics which are necessary for the MTO, transform with increasing TOS into more complex carbon residues called coke, resulting in a varying product selectivity over time and ultimately leads to a breakdown of the MTO due to pore blocking.^14^, ^19^

The application of ceramic membranes for in‐situ water removal was already considered for different reactions like the hydrogenation of CO_2_ 20 or pervaporation‐assisted esterification,^21^ mainly due to the increased conversion and changing product distribution caused by the water removal induced reaction equilibrium shift. However, in the case of the MTO reaction, the by‐product water could damage the catalyst hydrothermally as well.

As mentioned above, coking of the catalyst is a severe problem of the MTO process since it leads to pore blocking of the catalyst and severe diffusion problems.^22^, ^23^, ^24^, ^25^, ^26^, ^27^ Literature reports that the presence of steam in the feed should reduce catalyst coking, thus extending its lifetime.^28^, ^29^, ^30^, ^31^

The industrial application of the MTO reaction, a fluidized bed reactor, suffers heavily from attrition and requires the permanent exchange of the catalyst. In the used membrane reactor with fixed catalyst bed, the attrition problems are not existent, but the catalyst regeneration is more complex compared to the fluidized bed reactor.

## Results and Discussion

**Figure 1:**
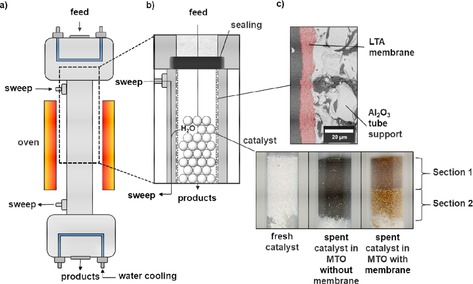
a) Membrane reactor setup for the MTO reaction at 450 °C. b) Magnification of the reactor inlet with the fixed bed of the SAPO‐34 catalyst (particle size 10 to 100 μm) inside the tubular LTA membrane. c) Cross section SEM image of LTA membrane on tubular α‐Al_2_O_3_ support and photos of the fresh SAPO‐34 catalyst, the spent catalyst after 250 min MTO reaction without membrane and the catalyst in the membrane reactor with water removal through the LTA tube membrane. Note that in case of the membrane supported MTO reaction, there is a sharp step in the color of the SAPO‐34 catalyst bed.

To further influence yield, selectivity and longevity of the catalyst and compare it with state of the art processes, we performed the MTO reaction in a membrane reactor with a SAPO‐34 packed bed catalyst, as shown in Figure 1. We have used the commercial SAPO‐34 catalyst developed for the MTO process by the Dalian Institute of Chemical Physics (DICP), which is described in detail elsewhere.^32^, ^33^, ^34^ In this paper, we have studied the influence of an in situ water removal through a hydrophilic membrane on the MTO reaction. The reaction is supported by a semi‐commercial hydrophilic Linde Type A (LTA) zeolite membrane, produced by the Fraunhofer IKTS Hermsdorf.^35^ The LTA membrane layer was grown on a tubular α‐Al_2_O_3_ support, to remove water in situ from the reaction environment.

**Figure 2:**
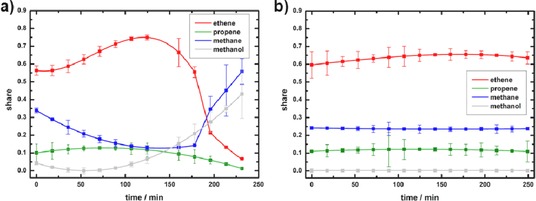
Development of the products (ethene, propene and methane) and non‐converted methanol in the MTO reaction at 450 °C over 250 minutes: a) without and b) supported by a LTA membrane.

Figure 1 a and b show the used membrane reactor setup. The MTO reaction was performed at 450 °C for 250 minutes (i) without a membrane and (ii) with in situ water removal through a tubular LTA membrane. The SAPO‐34 catalyst was piled on glass wool in the vertically oriented reactor. The SAPO‐34 catalyst changes its color from white in the beginning to black and brown as a result of the formation of high molecular weight hydrocarbon species inside the SAPO‐34 pores, often referred to as coke (Figure 1 c). However, while the catalyst without membrane support turns completely black, the SAPO‐34 catalyst bed, under in situ water removal through the LTA membrane, shows two sharply separated sections of different colors: A dark brown zone towards the reactor inlet, called Section 1, and a light brown zone in the direction of the reactor outlet, called Section 2. Later in this paper, we will analyze the carbonaceous depositions of these sections in detail. The main products (ethene, propene and methane) of the MTO reaction as well as non‐converted methanol, as determined by gas chromatography, are shown in Figure 2.

The MTO reaction on SAPO‐34 without membrane support exhibits changing product selectivity during the reaction and a significant deactivation of the catalyst after 180 minutes. The desired main products ethene (after 120 min) and propene (100 min) reach maxima in their selectivity at different points during the active period. Simultaneously, the conversion of methanol decreases and the concentration of methane strongly increases. On the other hand, the MTO reaction under continuous water removal through a LTA membrane shows a constant productivity of ethene, propene and methane over 250 minutes, without major fluctuations of the product concentrations. This is surprising, since we expected a decrease in the catalyst longevity due to the water removal. The reaction rate remains high, shown by the consistent low amount of unreacted methanol. The yield of light olefins does not exhibit any significant differences between the two measurement setups with and without membrane support. Compared to literature,^32^ we received similar results for an almost 100 % methanol conversion. Our DICP‐SAPO‐34 catalyst shows an exceptional high selectivity towards ethene, while the selectivity for propene and higher molecular alkenes is low. However, the product composition also depends, as mentioned above, on such experimental parameters like particle size and shape, reaction temperature, type of reactor and more. The LTA membrane itself without the SAPO‐34 catalyst showed almost no catalytic activity towards the MTO reaction.

A possible reason for the breakdown of the MTO reaction in the packed bed reactor without membrane support could be the hydrothermal damage of the SAPO‐34 catalyst due to steam. However, in our case the chabazite (CHA) crystal structure of SAPO‐34 stayed intact during the MTO reaction, as well as after regeneration by coke combustion in synthetic air at 500 °C, as the X‐ray diffraction patterns show (see SI Figure S1). The coked catalysts show a slight shift to lower diffraction angles compared to the fresh SAPO‐34 catalyst, indicating an expansion of the lattice due to the formation of larger hydrocarbon species inside the pores of the SAPO‐34. Therefore, the reason for the collapse of the MTO reaction after 3 h in the packed bed without membrane support seems to be solely the coking. The used SAPO‐34 is a silicoaluminophosphate of the CHA structure with 6.5×11 Å cages, connected through narrow 3.8×3.8 Å windows, consisting of 8‐membered oxygen rings, resulting in a 3D pore system.^36^, ^37^ The used catalyst has a reported acid density of 0.81 mmol/g.^34^ The different methanol reactions at the acidic sites will not only form light olefins but also a variety of high molecular weight hydrocarbon species in the pore system. Their formation and influence inside the pores of different acidic catalysts and their participation in the MTO reaction is a widely researched topic.^8^, ^15^, ^17^, ^22^, ^24^, ^36^, ^38^

**Figure 3:**
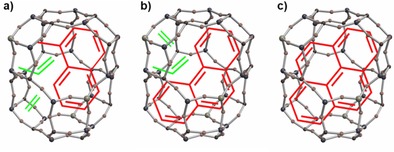
Schematic representation of the space requirements of the most common hydrocarbon species (see Figure 7) in a SAPO‐34 CHA cage, which build up in the course of the MTO reaction (red): a) methylnaphthalene, b) phenanthrene and c) pyrene. For the sake of comparison, the space requirements of the main products ethene and propene are shown (green), where they still fit additionally into the cage.

The induction period, starting with empty SAPO cages, is characterized by the sluggish kinetics of the formation of the first C−C bonds from methanol.^39^, ^40^ Eventually, they will transform into polymethylbenzenes inside the zeolite cages. The ongoing reaction of methanol ensures a high share of polymethylbenzenes with a high number of methyl groups (initiation phase). Studies with ^13^C‐labeled methanol showed the direct participation of the polymethylbenzenes in the formation of light olefins.^41^ The selective conversion of methanol to light olefins is set to take place in the so‐called working phase. It is assumed that in the working phase the penta‐ and hexamethylated arenes split off small alkenes, and turn into di‐ and trimethylated arenes. The re‐methylation and de‐alkylation of arenes is the essential mechanism of the light olefins formation.^14^, ^42^ The beneficial effects of the, in the MTO reaction participating, hydrocarbons recently led to the intentional and controlled formation prior to the reaction by “precoking”, which results in an enhanced ethene selectivity.^43^ However, with increasing TOS, the carbon species in the zeolite cages will build up and transform into naphthalenes, then phenanthrenes and ultimately pyrenes, the largest hydrocarbon species which will fit into the SAPO‐34 CHA cages. The space requirements of the most common hydrocarbons in the CHA cages of SAPO‐34 are schematically shown in Figure 3. The larger ones block the pores and acidic sites, resulting in a severely hindered mass transport (deactivated phase).^22^, ^44^ The medium sized aromatics in the cages lead to an improved light olefins formation in the initial TOS. Furthermore, the inherent structure of SAPO‐34 with its small windows prevents the diffusion of the arenes, as well as larger alkenes, enhancing the selectivity towards the light olefins even further.

**Figure 4:**
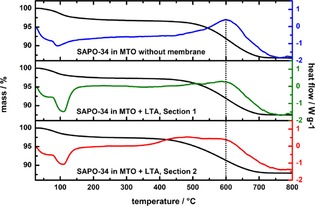
Thermogravimetry (black) and differential thermal analysis (colored) profiles of spent SAPO‐34 catalysts in a temperature range of 25 °C to 800 °C.

Surpringsly, the different thermogravimetric (TG) profiles (Figure 4) show similar contents of carbonaceous residues in the spent SAPO‐34 catalysts without and with membrane support (Sections 1 and 2, see Figure 1) of around 9 % (Table 1). However, the peaks in the differential thermal analysis (DTA) curves reflect different types of high molecular hydrocarbon species formed during the MTO reaction inside the SAPO‐34 CHA cages, indicating different stages of coking. The first type of hydrocarbon species formed is attributed to methylated benzene or naphthalene. Their decomposition temperature ranges between 400 °C and 500 °C. The DTA profile of Section 2 of the SAPO‐34 catalyst with the LTA membrane indicates a large presence of these types of hydrocarbons. Higher condensated and bulkier polyaromatics, combust at temperatures higher than 500 °C, peaking above 550 °C.^24^, ^38^ All types of catalyst (without and with LTA membrane Sections 1 and 2) have local maxima in the DTA curves at 600 °C (see dotted line, Figure 4) suggesting the existence of polyaromatics an all cases. Due to the observation that the catalyst without membrane support has a lower heat flow in the range between 400 and 500 °C, the percentage of larger polyaromatics is therefore higher compared to the catalysts+membrane.

**Table 1:** Mass loss due to carbon combustion in TG, decomposition temperatures of the high molecular hydrocarbon deposits (coke) in SAPO‐34 catalysts and BET surface area of fresh, coked and regenerated SAPO‐34, measured by nitrogen adsorption at 77 K. Coked samples after 250 minutes time on stream.

sample name	mass loss in carbon combustion step [%]	carbon combustion temp. [°C]	BET surface area [m^2^ g^−1^]
SAPO‐34	–	–	314.8
SAPO‐34 in MTO without membrane	9.3	539.8	31.1
SAPO‐34 in MTO+LTA, Section 1	9.1	529.1	45.2
SAPO‐34 in MTO+LTA, Section 2	9.0	488.5	46.4
SAPO‐34 in MTO after coke combustion	–	–	301.6

**Figure 5:**
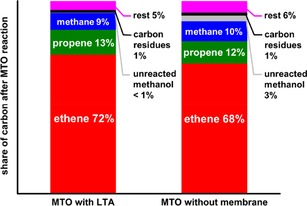
C‐balance: Share of carbon after performing the MTO reaction over the whole 250 minutes of reaction time with and without a water removal membrane.

The coking of the catalysts can additionally be seen by the decrease of the surface area, which was measured via BET nitrogen adsorption at 77.4 K. The results are summarized in Table 1. Due to the coke deposition, the surface area of SAPO‐34 without membrane was reduced by an order of magnitude compared to the fresh catalyst. However, in the case of membrane support, the reduction of the BET surface for similar coke contents is less severe. Though the BET surface areas of the coked SAPO‐34 from Sections 1 and 2 are very similar, they are still a third larger compared to the catalyst without membrane. By coke combustion, the surface area can be regenerated almost entirely, showing furthermore that there is no structural damage of the CHA structure due to the steam during the MTO reaction. Figure 5 displays the carbon balances of the two MTO reaction, indicating to which carbonaceous compound the carbon from the methanol source reacted. It summarizes the accumulated product over the whole 250 minutes of reaction time and gives similar results for the reaction with and without a LTA membrane. The course of the reaction, though, is more linear for the reaction with a LTA membrane, while the product composition of the MTO reaction without membrane fluctuates more (compare Figure 2).

For the most important points of the characteristic reaction course of the MTO reaction without membrane, we provide a similar study in the SI (Figure S4). The addition of an LTA membrane to the MTO reaction has no significant influence on the product composition after our defined reaction time of 250 minutes, as in both cases around 70 % of the carbon from the methanol reacts to ethene. However, the MTO reaction with the LTA membrane still works with the same efficiency, while the MTO reaction without membrane support collapsed. While the ethene yield of the membrane supported reaction should stay on the same level for even longer reaction times, the share of ethene without membrane support decreases due to the increasing amount of unreacted methanol. Moreover, the amount of carbon which reacts to the aforementioned carbon residues is slightly higher for the MTO reaction without membrane, but is located at around 1 % in both cases, see Figure 4 as well.

**Figure 6:**
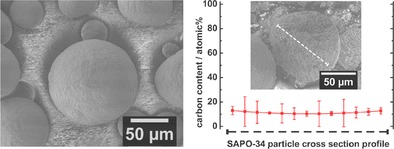
SEM pictures of coked SAPO‐34 catalyst after 250 min in MTO reaction at 450 °C. The particle size ranges between 10 and 100 μm, the individual SAPO‐34 crystal size is between 0.5 and 5 μm. Left: Outer surface of the spherical catalyst grain. Right: Coke content inside the broken SAPO‐34 particle according to EDXS. Inset: SEM image of broken SAPO‐34 particle, the direction of EDXS line scan path is shown (dotted line).

Figure 6 a) shows a SEM image of a spherical SAPO‐34 grain after the MTO reaction. The carbon content of a broken SAPO‐34 particle was determined via line scan EDXS (Figure 6 b) along the particle cross section. The results show that the amount of residual carbon inside the grain is dispersed relatively even over the whole particle. Subsequently, this shows again that the hydrocarbon species mainly form inside the catalyst and their desorption is heavily restricted by the small windows of the SAPO‐34 structure.

**Figure 7:**
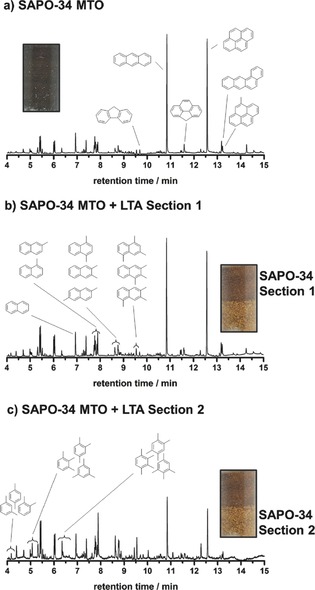
Gas chromatograms of retained hydrocarbon species in the CHA pores and/or deposited inside the catalyst grain of spent SAPO‐34 catalyst after 250 min in the MTO reaction at 450 °C: a) from the catalyst without membrane, b) from Section 1 and c) Section 2 of the catalyst with membrane‐support.

For the detailed characterization of the carbonaceous deposits (coke), gas chromatography‐mass spectrometry coupling (GC‐MS) has been applied. Therefore, a slightly modified extraction method, first introduced by Guisnet et al. was used to recover the confined hydrocarbon species from the SAPO‐34 cages.^45^ 15 mg of the spent catalyst were dissolved in 1.5 mL of 15 % HF. The organic compounds have been extracted with *n*‐hexane from the acidic solution. Subsequently, the analysis of the organic phase was carried out by GC‐MS, detailed information can be found in the supporting information. The analysis of the three types of spent catalysts (without membrane and with LTA membrane support Sections 1 and 2) are shown in Figure 7, the relative shares of the most common hydrocarbon species are summarized in Table 2. It becomes apparent that the three spent catalysts represent different stages of the MTO reaction. After 250 minutes of MTO reaction, the hydrocarbon species in the SAPO‐34 catalyst without membrane consists of over 55 % of bulky polyaromatics, like phenanthrene and pyrene. These large hydrocarbons block the pores and the catalytic active acidic sites, hinder diffusion and are responsible for the deactivation of the catalyst. These high molecular weight deposits explain the relative high combustion temperature in the TG profiles (Figure 4), as well as the low yield of light olefins after 250 minutes in the MTO reaction (Figure 2 a).

**Table 2:** Share of the most common retained high molecular hydrocarbon deposits in the CHA pores and/or inside the catalyst SAPO‐34 grains after 250 minutes time on stream.

hydrocarbon species	SAPO‐34 in MTO without membrane	SAPO‐34 in MTO+LTA, Section 1	SAPO‐34 in MTO+LTA, Section 2
methylated benzenes	6.6 %	11.5 %	20.7 %
naphthalene	6.6 %	7.5 %	9.3 %
methylated naphthalenes	16.2 %	21.6 %	36.9 %
phenanthrene/anthracene	30.8 %	24.4 %	15.8 %
pyrene	24.6 %	18.9 %	10.7 %

The GC‐MS results of section 1 of the SAPO‐34 catalyst with the membrane show a similar carbon composition like the catalyst without membrane support. However, the share of phenanthrene, anthracene and pyrene is only 43 %, the share of the medium sized aromatics, like methylated benzenes and naphthalene is therefore higher, compared to the catalyst without membrane. These medium sized aromatics do not hinder the diffusion of educts/products as much and can participate in the conversion of methanol to light olefins. While the SAPO‐34 catalyst without membrane is deactivated after 250 minutes, the catalyst in the membrane reactor is still active. Due to the presence of a high concentration of methylated benzenes and naphthalenes in Section 1 of the SAPO‐34 bed, we assume that this part of the bed membrane is still partially active. Section 2 of the bed has an even lower content of about 25 % of the bulky polyaromatic coke, while most of the hydrocarbon species are present as methylated smaller aromatics. Section 2 is, due to the high share of catalytically active hydrocarbon species, mainly working in the MTO reaction. The color changes of the catalyst are not necessarily connected to the contained aromatics, since the residues are all either colorless or light yellow. The different colors are the reason, why we decided to split the catalyst with membrane support into two sections in the first place and most likely stem from external coke, which does not play a significant role in the deactivation of the MTO reaction compared to the internal coke (hydrocarbon residues inside the SAPO‐34 pores).^11^

To further understand these findings, including the formation of the different Sections 1 and 2 of the catalyst bed inside the tubular LTA membrane, comparisons to existing models are required. Haw and Marcus proposed in 2005 a “cigar burn” mechanism to describe the development of the product selectivity of the MTO reaction in a SAPO‐34 catalyst bed, which is comparable to our approach of a membrane reactor with a packed catalyst bed.^42^ The reaction primarily takes place at the point where the methanol hits the catalyst first due to the high conversion rate of SAPO‐34.^24^ Consequently, the catalytically working zone (with methylated benzenes and naphthalenes) moves through the catalyst bed and leaves the deactivated catalyst (CHA cages completely occupied by pyrenes) behind. In our case, this working phase seems to be stabilized due to the water removal. We assume that this removal supports such reactions as the formation of the first C−C‐bonds and the methylation of aromatics, as Le Chatelier's principle would predict.

The formation of the first C−C‐bonds and, more importantly, the formation of the hydrocarbons from the HCP is more focused on the top of the moving working phase, whereas the aromatics inside the cages in the experiment without a membrane are more spread across the whole catalyst bed. As the catalyst bed inside the tubular LTA membrane developed two distinctive different sections, with different species of the carbonaceous deposits, the working phase is possibly a much narrower layer. Moreover, the methanol conversion rate stays high as well, due to the high amount of still active catalyst. On the contrary, the olefin production of the SAPO‐34 catalyst without membrane support changes during the reaction (Figure 2 a). First, it increases passing a maximum after 2 h and collapses after 3 h. Two mechanisms could cause this deactivation of the bed: (i) There is a homogeneous poisoning of the bed by coke, or (ii) an active zone like in the cigar burn mechanism moves faster, layer after layer, through the catalyst bed. The selectivity towards ethene increases with time caused by the formation of larger hydrocarbon species, due to steric limitations. The subsequent formation of even larger and bulkier hydrocarbons (coke) soon afterwards leads to the definite deactivation of the catalyst bed.

Improving selectivity and longevity of the MTO catalyst is a challenge to date. Control of the chemical composition of hydrocarbon deposits seems to be the key to fulfil this task, either by controlled preformation (“precoking”)^43^ or the influence of in‐situ methods like the shown continuous water removal through the LTA membrane.

Literature reports the beneficial impact of adding water to the feed on the longevity of the catalyst.^28^, ^29^, ^30^, ^31^, ^34^ These reports show a massive decrease in coke deposition due to the addition of water. In our experiment with similar reaction parameters, except for the addition of the LTA membrane, the removal of water also effects the longevity of the SAPO‐34 catalyst positively since – with the same amount of high‐molecular deposits as found by TG – in the case of the membrane reactor with steam removal these residues mainly consist of multi‐methylated benzenes. These methylated benzenes are believed to be active in the MTO reaction since they act as the main reaction intermediate, which will form ethylene by de‐alkylation as shown in.^11^ While the addition of water to the feed decreases the overall amount of coke produced, the removal of water primarily influences the composition.

We found that all types of post‐reaction catalyst (with and without membrane support) contain a similar amount of carbonaceous deposits, but their composition differs severely, even after they converted very similar amounts methanol to light olefins. Therefore, we conclude that the water removal seems to stabilize the catalytic active species (like methylated benzenes) longer, compared to the catalyst without membrane support. We believe the main reason is the assistance of the alkylation of the aromatics rings, where water is split off and can be immediately removed by the LTA membrane. Due to this equilibrium shift re‐alkylation and de‐alkylation may be favored compared to the condensation of the deposits to larger aromatic multi‐ring systems. This would maintain the catalytic active phase longer and explain why this phase moved slower trough the catalyst bed. Furthermore, water removal could benefit the re‐alkylation of the larger aromatic rings (like naphthalene and phenanthrene) as well, leading to a possibly increased participation of these larger aromatics in the formation of light olefins.

The product composition and development of the MTO reaction is influenced by a variety of reaction and material parameters, in particular the reactor type, the reaction temperature, the feed and sweep gas flow. Obviously, the catalyst type, the bulk density and its form and particle and crystal size distribution play the biggest role. We maintained these parameters for all of our experiments except for the addition of the LTA tube membrane, which conclusively led to the change of the carbonaceous deposits composition and the enhanced catalyst lifetime.

## Conclusions

We demonstrated the positive influence of removing water through an LTA zeolite membrane from a SAPO‐34 catalyst bed during MTO reaction in a tube membrane reactor. While the SAPO 34 catalyst without membrane deactivates after 3 hours, the SAPO 34 catalyst in combination with a water removing membrane maintains a steady conversion rate and selectivity over more than 4 hours. Thermogravimetry and GC‐MS confirmed that the reason for the collapse of the SAPO‐34 catalyst without membrane support is the pore blocking due to high molecular weight polyaromatics such as phenanthrene/ anthracene and pyrene, referred to as coke. In the membrane reactor, however, equal amounts of carbonaceous residues are found, but of less condensed rings such as methylated benzenes and naphthalenes. This type of residues is less likely to block acid sites and pore channels and even beneficial for the MTO reaction. Furthermore, the catalyst bed inside the tubular LTA membrane developed two distinctive different sections with different ratios of the various carbon residues species, called sections 1 and 2, which can additionally be distinguished by their colors. The catalyst bed near to the reactor inlet (section 1) contains a higher amount this high molecular weight polyaromatics and seems to be mostly deactivated. However, the lower section in the direction of the reactor outlet (section 2) contains mainly the catalytically active methylated benzenes and naphthalenes, which take part in the olefin production and give enough space for methanol conversion. We conclude that this zone, containing catalytically active hydrocarbons, moves through the catalyst bed, similar to the cigar burn mechanism. This motion of the coking zone is slower if water is removed since the re‐methylation of the aromatic rings inside the CHA cages is facilitated by the removal of water. Therefore, the water removal through the membrane supports the re‐methylation thus preventing the condensation of the aromatic rings to coke. In this study, we show that the fixed bed reactor setup in combination with a hydrophilic membrane could be an alternative to the existing fluidized bed reactor concept which suffers from an enormous attrition problem and requires, therefore, the permanent addition of new catalyst. Further research on this topic can be done by variation of several reaction parameters, such as temperature or particle size, as well as LTA pore size engineering by ion exchange.

## Experimental Section

The MTO reaction was performed at 450 °C for 250 minutes in a vertically orientated tube membrane reactor (Figure 1). The Al_2_O_3_ tube had a length of 300 mm and an outer diameter of 10 mm and was coated with a LTA zeolite membrane. The ceramic tube, respectively the reactor itself, were centrally filled with glass wool and 2 g of the SAPO‐34 catalyst was loosely piled on top, the catalyst was not chemically bounded to the LTA membrane. 50 ml/min of N_2_ were sent through a methanol reservoir, which was heated up to 50 °C and then directed into the tube reactor (WHSV=4.6 g g_cat_^−1^ h^−1^). To extract to retained hydrocarbon species from the spent catalyst, 15 mg of the respective spent SAPO‐34 was completely dissolved into 1.5 mL of 15 % HF for about 20 minutes. Afterwards, the HF solution was mixed with 1.5 mL of *n*‐hexane and left until the two phases had separated completely. The organic phase was removed and washed with saturated CaCl_2_ (in H_2_O) solution to remove possible HF remains. A detailed experimental section can be found in the supporting information.

## Conflict of interest

The authors declare no conflict of interest.

## Supporting information

As a service to our authors and readers, this journal provides supporting information supplied by the authors. Such materials are peer reviewed and may be re‐organized for online delivery, but are not copy‐edited or typeset. Technical support issues arising from supporting information (other than missing files) should be addressed to the authors.

Supplementary
