# Supplementary material for: Methanol‐to‐Olefins in a Membrane Reactor with in situ Steam Removal – The Decisive Role of Coking
Source: ChemCatChem. 2019 Nov 25;12(1):273–80. doi: 10.1002/cctc.201901222 (PMC7006748; doi:10.1002/cctc.201901222)
Supplement: Supplementary file 1 — Supplementary [file CCTC-12-273-s001.pdf]

CHEMCATCHEM

Supporting Information

© Copyright Wiley-VCH Verlag GmbH & Co. KGaA, 69451 Weinheim, 2019

Methanol-to-Olefins in a Membrane Reactor with in situ Steam Removal – The Decisive Role of Coking

Felix Rieck genannt Best,* Alexander Mundstock, Gerald Dräger, Pascal Rusch, Nadja C. Bigall, Hannes Richter, and Jürgen Caro*©2019 The Authors. Published by Wiley-VCH Verlag GmbH & Co. KGaA.
This is an open access article under the terms of the Creative Commons Attribution License, which permits use, distribution and reproduction in any medium, provided the original work is properly cited.

SUPPORTING INFORMATION

Methanol-to-Olefins in a Membrane Reactor with in situ Steam Removal – The Decisive Role of Coking

Felix Rieck genannt Best*[a], Alexander Mundstock[a], Gerald Dräger[b], Pascal Rusch[a], Nadja C. Bigall[a], Hannes Richter[c], Jürgen Caro*[a]

[a] Institute for Physical Chemistry and Electrochemistry, Leibniz University Hannover, 30167 Hannover, Germany

[b] Institute for Organic Chemistry, Leibniz University Hannover, 30167 Hannover, Germany

[c] Fraunhofer IKTS - Institute for Ceramic Technologies and Systems, 07629 Hermsdorf, Germany

Experimental Section

The MTO reaction was performed at 450°C for 250 minutes in a vertically orientated tube membrane reactor (**Fehler! Verweisquelle konnte nicht gefunden werden.**). The Al_2O_3 tube had a length of 300 mm and an outer diameter of 10 mm and was coated with a LTA zeolite membrane. The ceramic tube, respectively the reactor itself, were centrally filled with glass wool and 2 g of the SAPO-34 catalyst was suspended on top. 50 ml/min of N_2 were sent through a methanol reservoir, which was heated up to 50°C and then directed into the tube reactor (WHSV = 4.6 g g_{cat}^{-1} h^{-1}). The analysis of the MTO product composition was carried by gas chromatography on an Agilent GC 6890A, which was equipped with an Agilent 19095P-QO4, a Restek 19742 Q-Bond column and a thermal conductivity detector (TCD).
To extract to retained hydrocarbon species from the spent catalyst, 15 mg of the respective spent SAPO-34 was completely dissolved into 1.5 mL of 15% HF for about 20 minutes. Afterwards, the HF solution was mixed with 1.5 mL of *n*-hexane and left until the two phases had separated completely. The organic phase was removed and washed with saturated $CaCl_2$ (in H_2O) solution to remove possible HF remains. GC-MS analysis was performed on an Agilent 6890Plus GC with 5973N MSD using a Machery&Nagel Optima 5-MS capillary column (30 m*320 µm, 0.25 µm film; constant flow of 1.5 mL/min helium 5.0; 50 °C initial oven temperature with 1 min hold time, 20 °C/min gradient and 300 °C final temperature). EI mass spectra were recorded at 70 eV and analyzed based on the NIST spectral database.
X-Ray diffraction patterns, in the 2Θ range of 5° to 40°, were acquired on a Bruker D8 Advance diffractometer operating at 40 kV and 40 mA with Cu-Kα-radiation (λ = 0.154 nm).
The SEM images and EDXS measurements were acquired on a JEOL JSM 6700F operating at 10 kV and 10 µA with a LEI detector.
Thermogravimetric profiles were measured in air at a TGA/DSC 3+ from Mettler-Toledo in a temperature range between 20°C and 800°C with a heating rate of 15°C/min.

Results

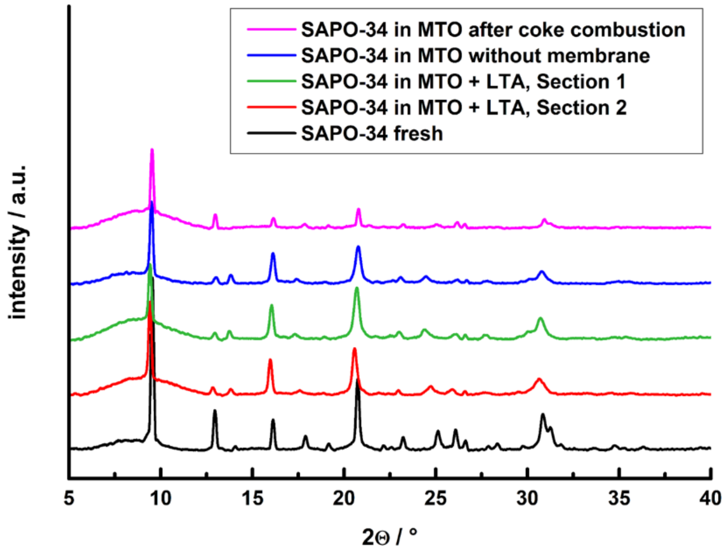

Figure S1. X-ray diffraction patterns of the fresh, spent and regenerated (via coke combustion at 500 °C in 40% oxygen/nitrogen stream for 4 h) SAPO-34 catalyst.

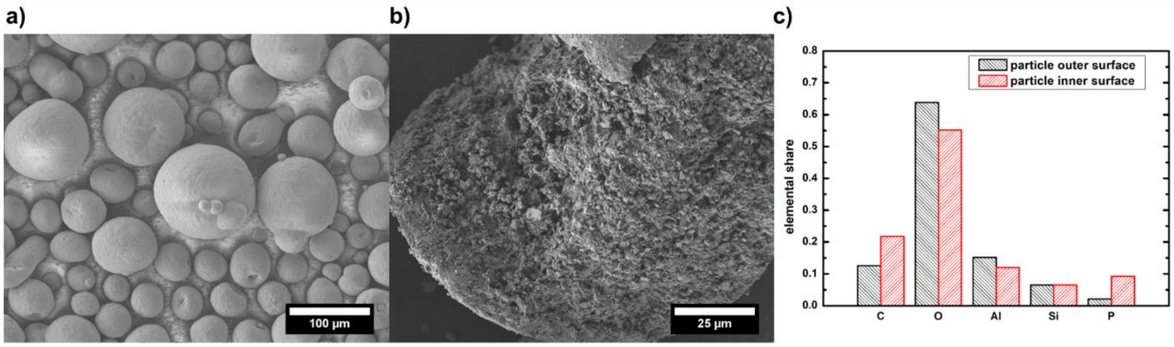

Figure S2. a) SEM image of the particle size distribution of the SAPO-34 catalyst, **b)** cross-section image and **c)** elemental distribution on the (i) inner and (ii) outer surface of SAPO-34 particle.

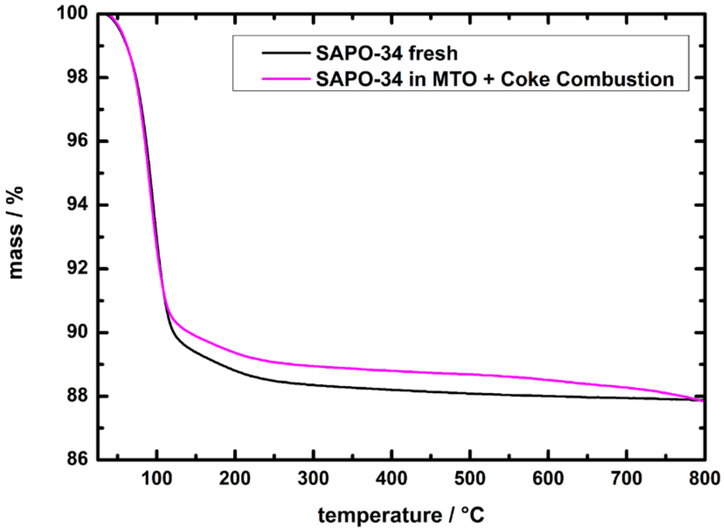

Figure S3. Thermogravimetry profiles of fresh and regenerated SAPO-34 catalysts in a temperature range of 20°C to 800°C.

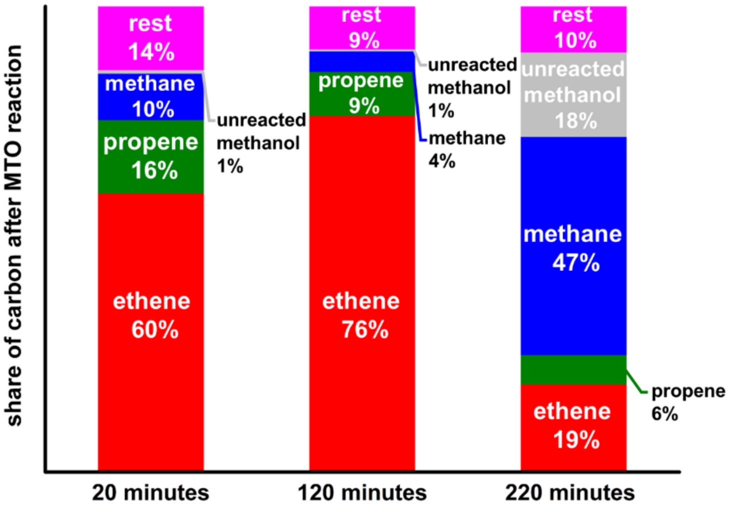

Figure S4. Share of carbon at selected points during the MTO reaction without membrane support.

Table 1. Extended analysis of retained hydrocarbons, extracted from the spent catalyst, via GC-MS.

hydrocarbon species	SAPO-34 in MTO without membrane	SAPO-34 in MTO + LTA, Section 1	SAPO-34 in MTO + LTA, Section 2
p-/*m*-/*o*-xylene	1.1%	4.5%	6.7%
1,2,4-/ 1,3,5-trimethylbenzene	3.8%	5.8%	8.6%
1,2,3,5-tetramethylbenzene	1.7%	1.3%	5.4%
naphthalene	6.6%	7.5%	9.3%
1,3-di-*tert*-butylbenzene	3.8%	5.0%	6.6%
1-/ 2-methylnaphthalene	9.8%	11.9%	21.3%
cyclopentane	2.4%	2.6%	0.0%
1,3-/ 2,3-/ 2,7-/… dimethylnaphtha-lene	5.2%	6.8%	10.2%
1,6,7-/ 1,4,6-/… trimethylnaphtale-ne	1.3%	1.8%	5.4%
fluorene	1.4%	1.7%	0.0%
phenantrene/ anthracene	30.8%	24.4%	15.8%
4H-cyclopenta[*def*]phenanthrene	2.3%	3.2%	0.0%
11H-benzo[*b*]fluorene	2.3%	0.8%	0.0%
pyrene	24.6%	18.9%	10.65%
benzanthracene	1.9%	1.6%	0.0%
1-/ 4- methylpyrene	1.15	1.4%	0.0%
